# Are statins a promising therapeutic strategy for macular edema? Insights from a systematic review and meta-analysis

**DOI:** 10.1186/s40942-025-00724-y

**Published:** 2025-11-04

**Authors:** Kai-Yang Chen, Hoi-Chun Chan, Chi-Ming Chan

**Affiliations:** 1 Department of General Medicine, Chang Gung Memorial Hospital (Linkou Branch), Taoyuan, Taiwan; 2 School of Pharmacy, China Medical University, Taichung, Taiwan; 3 Department of Ophthalmology, Cardinal Tien Hospital, New Taipei City, Taiwan; 4 School of Medicine, Fu Jen Catholic University, New Taipei City, Taiwan

**Keywords:** Statins, Diabetic macular edema (DME), Visual acuity, Dyslipidemia

## Abstract

**Introduction:**

Macular edema, a major cause of vision loss in diabetic retinopathy, involves retinal fluid accumulation and retinal thickening due to vascular dysfunction and inflammation. Standard treatments include anti-VEGF agents, corticosteroids, and laser photocoagulation, but limitations such as incomplete response and adverse effects exist. Statins, known for lipid-lowering and pleiotropic anti-inflammatory effects, have emerged as a potential therapeutic option.

**Methods:**

We conducted a systematic review and meta-analysis registered with PROSPERO (CRD420251082672) following PRISMA guidelines. Peer-reviewed studies evaluating statins’ efficacy and safety in macular edema were identified via multiple databases up to May 2025. Included studies encompassed randomized controlled trials and observational studies involving patients with macular edema treated with statins versus controls. Key outcomes were hard exudate reduction, retinal thickness, visual acuity, diabetic retinopathy progression, and safety.

**Results:**

Ten eligible studies involving atorvastatin and simvastatin showed statins significantly reduced hard exudates (risk ratio 4.42, p=0.03) and retinal thickness, with a 58% reduction in diabetic retinopathy progression. Statins also decreased the need for laser treatment by 32%. Visual acuity improvement was inconsistent, with benefits mainly in dyslipidemic patients. Safety analysis revealed no significant increase in adverse events. Statin effects were more pronounced in patients with elevated lipid levels.

**Conclusion:**

Statins demonstrate promising anatomical and preventive benefits in diabetic macular edema, particularly in patients with dyslipidemia, by reducing lipid exudation and stabilizing retinal vasculature. Despite limited visual acuity improvement, their favorable safety profile supports considering statins as adjunctive therapy. Further large-scale, long-term trials are warranted to confirm these findings and optimize treatment protocols.

**Supplementary Information:**

The online version contains supplementary material available at 10.1186/s40942-025-00724-y.

## Introduction

Macular edema is a challenging-to-manage ophthalmic condition characterized by an abnormal accumulation of fluid within the macula [1]. The resultant fluid retention, through its pathological mechanism, results in retinal thickening and the formation of cystoid spaces that disrupt the photoreceptor layer [2, 3]. Various pathological conditions can cause macular edema, including retinal vein occlusions (RVO), uveitis, postoperative inflammation, and age-related macular degeneration [4–6]. In addition, diabetic retinopathy is associated with macular edema. DME occurs in about 7% of diabetic patients and is the most common cause of vision loss [7]. The diseases share underlying pathophysiologic mechanisms, including vascular endothelial dysfunction, inflammatory cascades, and blood-retinal barrier disruption, leading to macular extracellular fluid accumulation [3, 8]. Macular edema can cause vision impairment, from distortion to vision loss [4]. In addition, chronic edema can cause irreversible photoreceptor damage and permanent visual loss [9].

There are several standard treatments used as intervention of Macular edema. Nevertheless, statins have emerged as a promising therapeutic option for macular edema due to their pleiotropic effects. Statins can reduce inflammation through cytokine suppression and NF-κB inhibition [10], enhancing endothelial function and blood-retinal barrier stability via nitric oxide modulation [11]. They also reduce oxidative stress through NADPH oxidase inhibition [12] addressing the core contributors to fluid accumulation and retinal thickening.

Current standard treatments for macular edema include intravitreal anti-vascular endothelial growth factor (anti-VEGF) agents, corticosteroids, and focal/grid laser photocoagulation [13]. While anti-VEGF therapy has revolutionized macular edema management, particularly for diabetic and RVO-associated edema, several limitations persist [14]. Approximately 40% of patients demonstrate incomplete response to anti-VEGF agents, requiring frequent injections that pose cumulative risks of endophthalmitis, retinal detachment, and vitreous hemorrhage [15, 16]. Corticosteroid treatments, while effective, are associated with significant ocular adverse effects, including cataract formation and intraocular pressure elevation [17]. Laser photocoagulation, though providing stabilization, rarely significantly improves vision and carries risks of scotoma formation and progressive visual field loss [18, 19]. The limitations of these treatment approaches highlight the need for alternative or adjunctive treatment modalities with favorable efficacy and safety profiles.

Statins (3-hydroxy-3-methylglutaryl coenzyme A reductase inhibitors) are primarily recognized as lipid-lowering agents that reduce cardiovascular morbidity and mortality by inhibiting cholesterol biosynthesis [20, 21]. Due to their favorable safety profile, statins are increasingly used in clinical practice [22]. In addition, they exhibit pleiotropic effects, including lipid metabolism, anti-inflammatory actions, antioxidant effects, and endothelial stabilization [23]. In addition, they regulate inflammatory responses by suppressing proinflammatory cytokine expression, preventing leukocyte-endothelial interactions, and reducing microglial activation [24]. Their antioxidant effect inhibits oxidative stress, increases endothelial nitric oxide synthase activity, and decreases endothelin-1 expression, thus improving endothelial function and repairing blood-retinal barrier integrity [24–27]. Statins also lower VEGF expression levels, a key vascular permeability mediator in retinal disease, complementing anti-VEGF therapeutics [28].

Despite the reported advantages of statins in macular edema therapy, clinical evidence for their efficacy is inconclusive [29]. The currently existing literature includes narrative summaries or isolated observational studies, which vary widely in design, patient populations, and outcome measures [30, 31] This lack of consolidated evidence underscores the need for a rigorous systematic review and meta-analysis to evaluate statins’ therapeutic potential in macular edema. Furthermore, the heterogeneity in the designs of most trials, populations enrolled by these trials, their dosing regimens, and the outcome measures contributes to these inconsistencies in findings. Therefore, this study will evaluate statins’ efficacy and safety profile in managing macular edema by synthesizing available evidence.

### Research objectives

To evaluate statin therapy’s efficacy and safety profile in treating and preventing macular edema.

### Research question

How effective are statins in reducing hard exudates, improving retinal thickness, and enhancing visual outcomes in patients with DME?

How do baseline lipid profiles influence the therapeutic response to statin therapy in patients with macular edema?

### Methodology

The reporting of this study adhered to the Preferred Reporting Items for Systematic Reviews and Meta-Analysis (PRISMA) [32]. Our systematic review has been registered on an online registration website, PROSPERO, the number is (CRD420251082672).

### Identification and selection of studies

A reviewer (K.Y.C.) conducted a comprehensive literature search for peer-reviewed original research articles via PubMed, EBSCO Open Research, ScienceDirect, and Wiley Online Library from inception to May 1st, 2025. In addition, the first 20 pages of Google Scholar were manually searched for relevant research articles.

### Search strategy

Keywords were identified from a preliminary search conducted by a reviewer (K.Y.C.). The keywords were combined using Boolean operators and used with MeSH terms to formulate search strings (Table 1). The keywords were pilot tested via the databases for their reliability to identify relevant research articles on the safety and efficacy of statins in macular edema management. A reviewer (K.Y.C.) then conducted a comprehensive database search. The searching strategy includes: (Statins OR “HMG-CoA reductase inhibitors” OR Atorvastatin OR Simvastatin OR Rosuvastatin OR Pravastatin OR Lovastatin OR Fluvastatin OR Pitavastatin OR Cerivastatin) AND (“Retinal edema” OR “Intraretinal fluid” OR “Cystoid macular edema” OR “Diabetic macular edema” OR “Pseudophakic macular edema” OR “macular edema” OR “Serous macular detachment” OR “Chronic macular edema” OR “Refractory macular edema”).

**Table 1:** Identified keywords

Statins	Macular Edema
(Statins OR “HMG-CoA reductase inhibitors” OR Atorvastatin OR Simvastatin OR Rosuvastatin OR Pravastatin OR Lovastatin OR Fluvastatin OR Pitavastatin OR Cerivastatin)	(“Retinal edema” OR “Intraretinal fluid” OR “Cystoid macular edema” OR “Diabetic macular edema” OR “Pseudophakic macular edema” OR “macular edema” OR “Serous macular detachment” OR “Chronic macular edema” OR “Refractory macular edema”)

### Study selection

The retrieved results were exported to Zotero screening software version 6.0.36. The software automatically identified ineligible and duplicate records by comparing metadata, which a reviewer (K.Y.C.) manually merged. The reviewer then screened the remaining articles.

Two independent reviewers (K.Y.C. and H.C.C.) screened the non-duplicate records by titles and abstracts, followed by full texts, against the prespecified eligibility criteria.

### Eligibility criteria

#### Inclusion criteria

This study included research on the safety and efficacy of statins in macular edema management. The PICOS criteria were used to select the studies [33].

The PICOS criteria for eligible studies were defined as follows;

Population (P): Patients with macular edema.

Intervention (I): Statins therapy, including atorvastatin and simvastatin.

Comparison (C): Control groups receiving standard care without statins, placebo, or conventional treatment protocols.

Primary outcomes (O): reduction of hard exudate, retinal thickness changes, visual acuity, safety profile (ocular and systemic adverse events), disease progression markers, and preventive effects on DME occurrence. The quantification of hard exudate is done using fundus imaging in trials like the ACCORD Eye Study and other DME-focused interventions. This method is recognized as a valid surrogate for lipid leakage in the retina [34].

Study Design (S): RCTs, cohort studies, case-control studies, cross-sectional studies, and other suitable study designs investigating statin efficacy and safety in treating and preventing macular edema.

#### Exclusion criteria

Case reports, narrative reviews without original data, non-peer-reviewed abstracts or gray literature, animal/in vitro studies without clinical relevance, and non-English publications were excluded. In addition, studies using non-statin lipid-lowering agents, lacking clear statin dosage/regimen, or combining therapies without isolating statin effects were excluded.

#### Methodological quality assessment

Two independent reviewers (K.Y.C. and H.C.C.) assessed the methodological quality of the included studies. The RoB 2.0 tool was used to assess the risk of bias in two RCTs, evaluating bias from the randomization process, deviations from intended interventions, missing outcome data, outcome measurement, and selection of the reported result [35]. For non-randomized studies, the ROBINS-I tool was used to assess bias due to confounding, selection of participants, classification of interventions, deviations from intended interventions, missing data, measurement of outcomes, and selection of reported results [35]. Discrepancies were resolved through discussion or by a third reviewer (C.M.C.) when needed.

#### Data selection and extraction

Data from all eligible studies were systematically extracted and organized using Microsoft Excel 2019. The data extraction process involved a thorough review of each study’s full text by two independent reviewers (K.Y.C. and H.C.C.), with discrepancies resolved through discussion or a third reviewer (C.M.C.) when necessary. The following data sets were extracted: study ID, country, study design, population characteristics, type of macular edema, intervention details, outcomes measured, efficacy outcomes, safety outcomes, objectives, and findings.

#### Data analysis

The extracted data from the studies were thematically analyzed according to the outcome measures of interest [36]. In addition, quantitative data were analyzed using Review Manager software version 5.4.1. An intervention review starting from the full review stage was used. Dichotomous data types were used, applying the Mantel-Haenszel statistical method and a random effects analysis model with odds ratio effect measure.

## Results

### Study selection

**Fig. 1:**
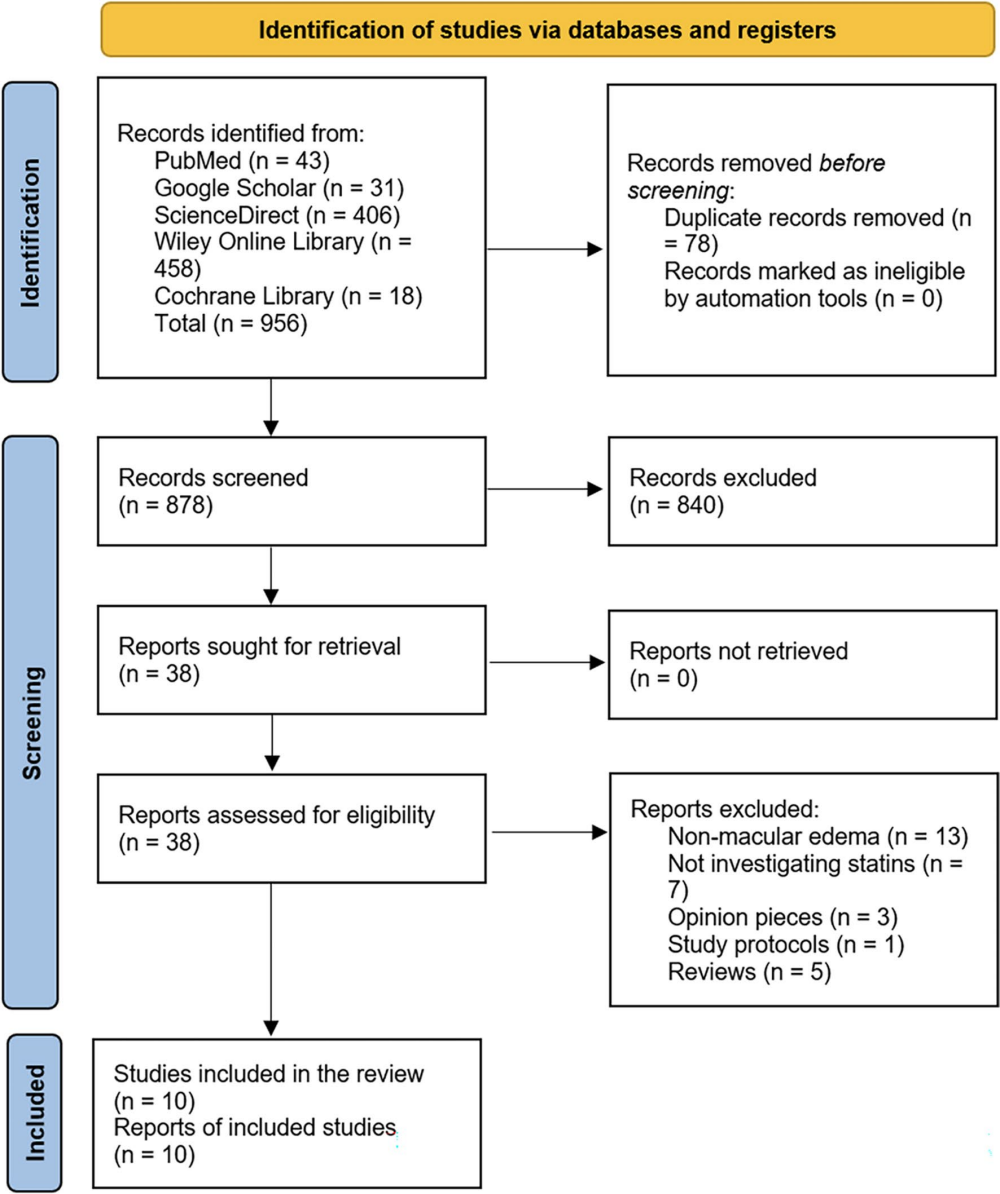
PRISMA flowchart for study selection process

The PRISMA flowchart below (Fig. 1) outlines the process of identifying, screening, and including studies in this systematic review. The online search for studies resulted in a total of 956 records initially identified through various databases, including 43 from PubMed, 31 from Google Scholar, 406 from ScienceDirect, 458 from Wiley Online Library, and 18 from the Cochrane Library. Before screening, 78 duplicate records were removed, and no records were marked ineligible by automation tools, leaving 878 records to be screened. During the screening phase, 840 records were excluded based on title and abstract. The remaining 38 reports were sought for retrieval, and all were successfully retrieved. These 38 reports were assessed for eligibility, out of which 28 were excluded for various reasons: 13 did not focus on macular edema, 7 did not investigate statins, 3 were opinion pieces, 1 was a study protocol, and 5 were reviews. Ultimately, 10 studies were included in the systematic review, with 10 corresponding reports forming the final evidence base (Fig. 1).

### Methodological quality assessment

**Fig. 2:**
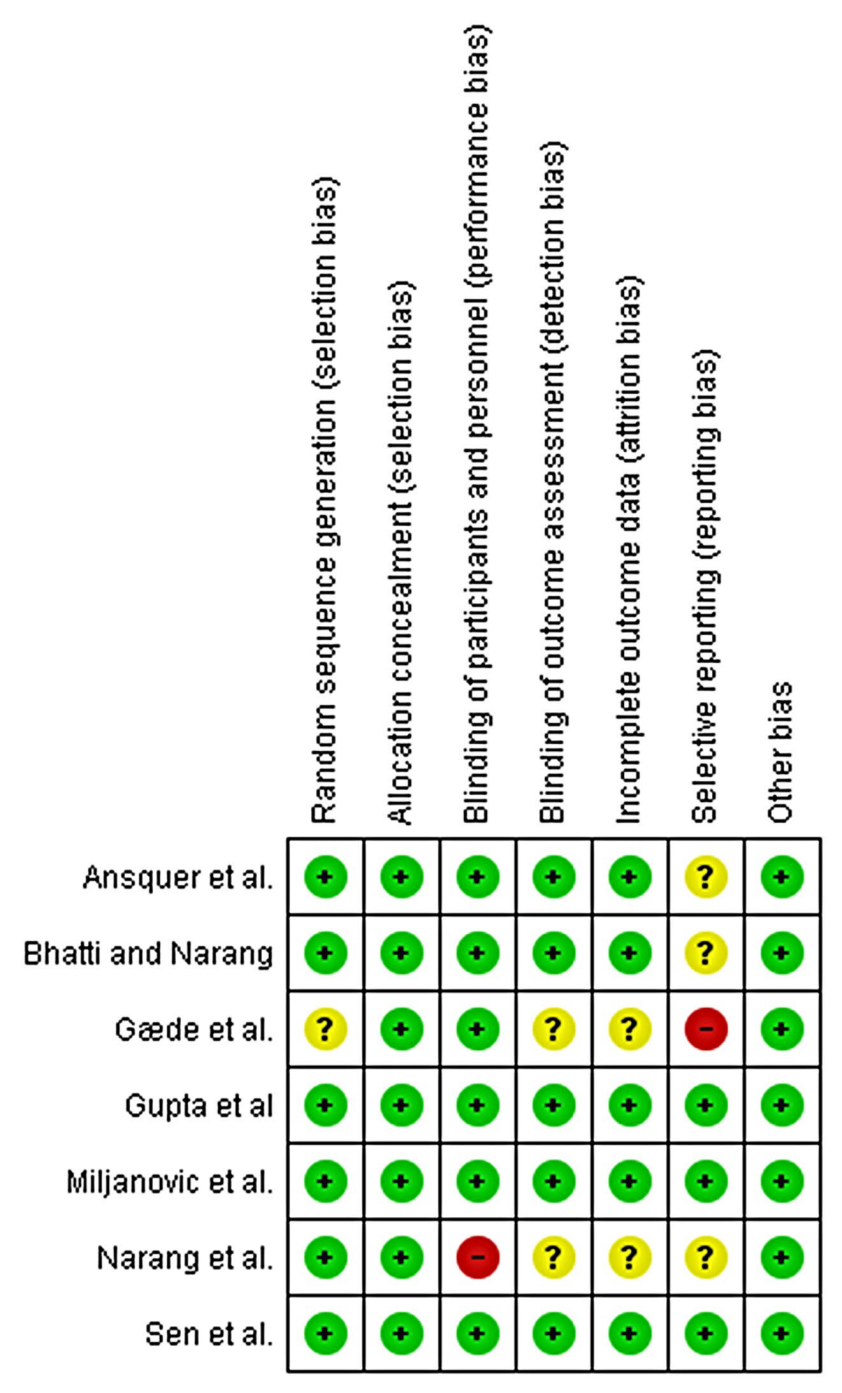
Table of the risk of bias assessment of RCT studies using ROB 2.0 tool

**Fig. 3:**
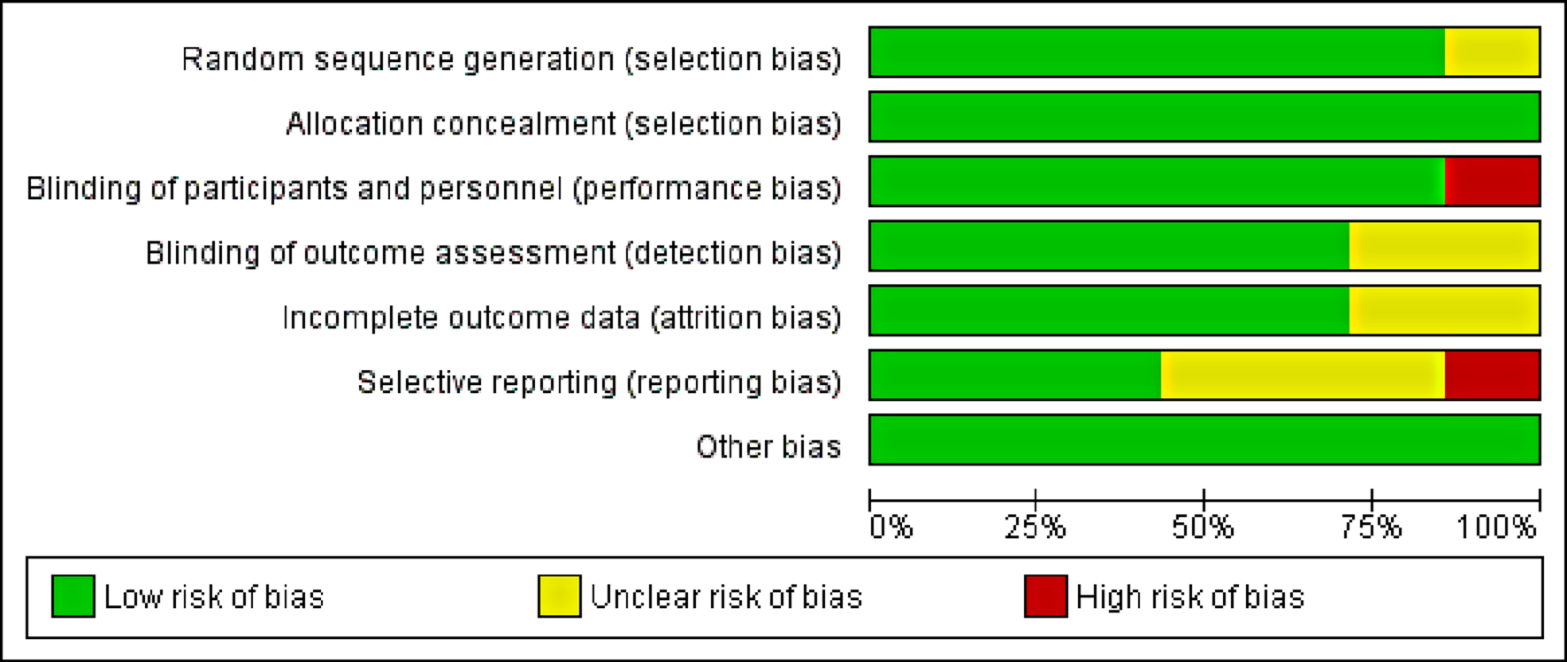
Graph of the summary of risk of bias assessment for RCT studies using ROB 2.0 tool

The Risk of Bias assessment using the RoB 2.0 and ROBINS-I tool revealed a low risk of bias arising from the randomization process, deviations from intended interventions, missing outcome data, measurement of outcomes, confounding, selection of participants, classification of interventions, and selection of reported results (Figs. 2 and 3) and (Table 2).

**Table 2:** Results of ROBINS I risk of bias

Study	Bias due to Confounding	Bias in Selection of Participants	Bias in Classification of Interventions	Bias due to Deviations from Intended Interventions	Bias due to Missing Data	Bias in Measurement of Outcomes	Bias in Selection of the Reported Result	Overall Risk of Bias
Chung et al. (2017)	Moderate: Potential confounders like age and comorbidities were not fully adjusted for.	Moderate: Limited control over selection bias.	Low: Statin therapy clearly defined.	Low: Statin therapy is the primary intervention.	Low: A clear methodology for patient inclusion	Low: Established outcomes measurement methods	Moderate: Limited exploration of other potential variables.	Moderate
Panagiotoglou et al. (2010)	High: No control for confounding factors.	High: No randomization or blinding, leading to selection bias.	Low: Clear distinction between statin and non-statin groups.	Low: Clear distinction in intervention between groups.	Low: No major missing data issues.	Low: Fluorescein leakage measured reliably.	Low: Focus on relevant clinical outcomes, no evidence of selective reporting.	High
Ucğun et al. (2007)	Low: The study has controls for some key variables (e.g., lipid profiles, HbA1c)	The study uses a group-based comparison of patients with and without exudative diabetic macular edema (EDME).	Low: There is a well defined classification of patients.	Low: No deviations from the intended intervention.	Low: The study reports complete data for both groups	The study uses standard clinical measurements of serum lipid levels and HbA1c	Low: The outcome of interest is clearly defined and consistently measured across groups.	Low

### Data selection and extraction

This analysis included 6 RCTs, two cohort studies, and a case series conducted across the USA, India, South Korea, Denmark, Turkey, and China. The studies examined statins and fibrates in diabetic patients with different types of macular edema. Sample sizes ranged from 18 to 1,441 participants. In addition, primary interventions included atorvastatin, simvastatin, fenofibrate, and multifactorial approaches. Key outcomes measured were visual acuity changes, hard exudate reduction, macular oedema resolution, retinopathy progression, and need for laser treatment (Table 3).

**Table 3:** Study characteristics

Study	Country	Study design	Population Characteristics	Type of Macular Edema	Drugs Administered	Intervention Details	Outcomes Measured Efficacy Outcomes	Safety Outcomes:	Objectives	Findings
Ansquer et al. (2011)	USA	RCT	Unspecified	DME	Beta-Carotene, alpha-Lipoic acid, N-Acetylcysteine (NAC), Aminoguanidine, Curcumin, Pycnogenol, Taurine, Zinc	Lipid-lowering drugs vs. placebo (comparison of lipid-lowering drugs)	Need for laser treatment Retinopathy progression Macular oedema development	Not explicitly reported	Evaluate the effects of lipid-lowering drugs	Fenofibrate reduced laser treatment for DME/PDR by 31% (3.4% vs. 4.9%, *p* < 0.001) Composite endpoint reduced by 34% (11.1% vs. 16.1%, *p* = 0.022) Statins alone did not show similar benefits.
Bhatti and Narang (2020)	India	RCT	50 type 2 diabetic patients.	Clinically significant macular edema (CSME)	Atorvastatin **Dosage**: 10–20 mg/day **Period**: 1,3 and 6 months	Atorvastatin (10–20 mg/day) for ≥ 6 months	Change in hard exudate grade (ETDRS grading) Maximum retinal thickness (MRT) on OCT Visual acuity (logMAR)	Not explicitly reported	Evaluate the effect of atorvastatin on hard exudates and CSME in type 2 diabetics with a normal lipid profile.	Hard exudates: 68% improved by 1 grade, 4% by two grades; 24% stable, 4% worsened MRT: Significant reduction (360.03 ± 30.94 μm to 284.6 ± 48.3 μm, *p* < 0.001) Visual acuity: No significant change (0.73 ± 0.4 to 0.76 ± 0.4 logMAR, *p* = 0.92)
Chung et al. (2017)	South Korea	Retrospective cohort study	110 type 2 diabetic patients. Statin users (*n* = 70): Older (58.1 ± 11.6 yrs), longer diabetes duration (12.4 ± 8.0 yrs) Non-users (*n* = 40): Younger (52.3 ± 12.2 yrs), shorter diabetes duration (8.6 ± 8.2 yrs)	DME with central retinal thickness ≥ 300 μm (OCT-confirmed)	Specific statin and dosage not define **Period**: 6 months	Statin therapy (type/dosage not specified) vs. no statin use	DR progression (≥ 2 ETDRS steps) DME incidence (OCT-based)	Not explicitly Reported	Investigate the effects of statins and dyslipidemia on DR progression and DME in type 2 diabetics.	DR progression: No significant difference (23% statin users vs. 18% non-users, *p*=0.506) DME incidence: Lower in statin users (23% vs. 48%, *p*=0.008) - Lipid profiles: Statin users had lower LDL (*p*=0.007). DME patients had higher triglycerides (*p*=0.004) and lower HDL (*p*=0.033) Logistic regression: Statins reduced DME risk (OR = 0.33, 95% CI 0.12–0.91, *p*=0.032). Hypertriglyceridemia predicted DME (OR = 1.52, 95% CI 1.14–2.02, *p*=0.005)
Gæde et al. (2008)	Denmark	RCT	160 type 2 diabetic patients Mean age: ~55 years Intensive therapy (*n* = 80) vs. conventional therapy (*n* = 80)	DME	Statins (85% usage), RAS blockers, aspirin, glycemic/lipid/BP **Period**: 5.5 months (averaged)	Intensive therapy: Multifactorial intervention including statins (85% usage), RAS blockers, aspirin, glycemic/lipid/BP control Conventional therapy: Standard care (22% statin usage initially)	Retinopathy progression Need for retinal photocoagulation (for proliferative retinopathy/macular edema)	Minor side effects (e.g., muscle pain with statins)	Evaluate effects of intensive multifactorial therapy (including statins) on mortality and microvascular complications (including retinopathy/macular edema)	Retinal photocoagulation: Fewer in intensive group (14 vs. 27 patients; RR = 0.45, 95% CI 0.23–0.86, *p*=0.02) Retinopathy progression: Reduced in intensive group (RR = 0.57, 95% CI 0.37–0.88, *p*=0.01) Safety: No major statin-related adverse events (1 case of muscle pain)
Gupta et al. (2004)	India	RCT	30 type 2 diabetic patients with CSME and dyslipidemia (21 male, 9 female)	Clinically significant macular edema (CSME) with hard exudates	Atorvastatin **Dosage**: 10 mg/day 3-hydroxy-3-methylglutaryl coenzyme A inhibitor, laser photocoagulation **Period**: 18 weeks	Group A (*n* = 15): Atorvastatin (10 mg/day, titrated to target LDL < 100 mg/dL) + laser photocoagulation Group B (*n* = 15): Laser photocoagulation alone (no statin)	Reduction in hard exudates, Subfoveal lipid migration, Macular edema regression, Visual acuity	Not explicitly Reported	Evaluate atorvastatin’s efficacy in reducing hard exudates and subfoveal lipid migration post-laser in diabetic CSME with dyslipidemia.	Hard exudates: Improved in 66.6% (10/15) statin vs. 13.3% (2/15) control (*P* = 0.007) Subfoveal lipid migration: 0% statin vs. 33.3% (5/15) control (*P* = 0.04) Macular edema regression: 60% (9/15) statin vs. 33% (5/15) control (*P* = 0.27) Visual acuity: Worsened in 0% statin vs. 20% (3/15) control (*P* = 0.22)
Miljanovic et al. (2004)	USA	RCT	1,441 type 1 diabetic patients (aged 13–39 years, diabetes duration 1–15 years)	Clinically significant macular edema (CSME)	Atorvastatin	Atorvastatin	CSME incidence Hard exudate development	Not explicitly Reported	Examine relationships between serum lipid levels and diabetic retinopathy outcomes.	Higher LDL and total: HDL ratio predicted CSME (RR 1.95 and 3.84, respectively, both *p* = 0.03) Same lipids predicted hard exudates (RR 2.77 and 2.44, respectively, *p* ≤ 0.002)
Narang et al. (2012)	India	RCT	30 patients with CSME and a normal lipid profile	Clinically significant macular edema (CSME)	Atorvastatin **Dosage**: 20 mg/day **Period**: 6 months	Group A (*n* = 15): Atorvastatin 20 mg/day starting 4 weeks pre-laser Group B (*n* = 15): Placebo	Visual acuity change Macular edema resolution Hard exudate changes	Liver function tests, CPK-MM	Evaluate atorvastatin’s effect on CSME in people with diabetes with normal lipid profiles.	No significant difference in visual outcomes (100% vs. 86.67% success, *p* = 0.14) No difference in macular edema resolution (86.67% vs. 80%, *p* = 0.62) Hard exudate improvement (46.66% vs. 33.33%, *p* > 0.1)
Panagiotoglou et al. (2010)	USA	Case series	18 Patients	DME	Atorvastatin **Period**: 12 months	Atorvastatin	Hard exudates Fluorescein leakage Hemorrhages	Not mentioned	Determine the efficacy of atorvastatin in reducing hard exudates and DME	Significant decrease in total cholesterol and LDL (*P* < 0.05) Reduction in hard exudates and fluorescein leakage No change in hemorrhage status
Sen et al. (2002)	China	RCT	50 diabetic patients (Type 1 & 2)	Non-clinically significant macular edema	Simvastatin **Dosage**: 20 mg/day **Period**: 180 days	Simvastatin 20 mg/day vs. placebo for 180 days	Visual acuity Retinopathy progression (FFA & fundus photography)	Not mentioned	Evaluate simvastatin’s effect on macular edema progression	Significant lipid reduction with simvastatin (TC, LDL-C *P* < 0.001; HDL-C increase *P* < 0.001) VA improved in 4 simvastatin vs. zero placebo patients (NS) Worsening VA: 0 simvastatin vs. 7 placebo (*P* = 0.009) Retinopathy progression: 1 improved (simvastatin) vs. 7 worsened (placebo) (*P* = 0.009)
Ucgun et al. (2007)	Turkey	Retrospective cohort study	54 type 2 diabetic patients with NPDR Group A: 27 with exudative macular Group B: 27 without macular edema Excluded: Renal/liver/heart disease	Exudative DME	Simvastatin	Simvastatin	Lipid profile comparison. HbA1c levels	Not mentioned	Evaluate the relationship between serum lipids and exudative diabetic maculopathy.	Higher cholesterol (224.30 vs. 197.78 mg/dL, *P* = 0.038) Higher LDL (150.59 vs. 124.37 mg/dL, *P* = 0.026) Higher HbA1c (9.62 vs. 7.36 g/dL, *P* = 0.000) in macular edema group

### Thematic analysis of outcomes

#### Efficacy of statins

Statins showed clinically meaningful benefits for DME. Atorvastatin intervention reduced hard exudates by 66.6%, slightly better than the 13·3% reduction in the control group [37]. Hard exudates and fluorescein leakage were decreased after atorvastatin intervention use in diabetic maculopathy patients, and a significant decrease in total cholesterol and low-density lipoprotein cholesterol was recorded [38]. Similarly, structural improvement was observed, evidenced by a significant reduction in the maximum retinal thickness from 360.03 ± 30.94 μm at the baseline to 284.6 ± 48.3 μm following treatment (*p* < 0.001) [39]. Simvastatin exhibited protective effects against vision loss, with no patients experiencing worsening visual acuity compared to 28% in the placebo group (*p* = 0.009) [40]. However, atorvastatin did not express significant visual benefits in patients with normal lipid profiles for visual acuity outcomes (*p* = 0.14) (Narang et al., 2012). The effect size for hard exudate improvement in dyslipidemic patients was quite significant, with an OR of 12.5 [37].

The therapeutic response to statins showed dependence on lipid levels. Patients with elevated lipids showed better results, as demonstrated by the correlation between LDL reduction and clinical improvement (*p* < 0.001) [40]. In contrast, normolipidemic patients recorded minimal improvement after treatment, with nonsignificant differences in both the visual acuity (*p* = 0.14) and macular edema resolution (*p* = 0.62) between the treatment and control groups [41]. The small inconsistencies noticed in the measure of visual acuity arise from the differences in study designs, including randomized controlled trials (RCTs) and observational studies, since observational studies are more prone to bias and confounding factors. Nevertheless, the inconsistent outcome measures, such as varying OCT parameters and grading scales for visual acuity, added to the variability in results. Finally, differences in participant demographics, disease severity, and comorbid conditions also likely impacted treatment responses.

**Fig. 4:**
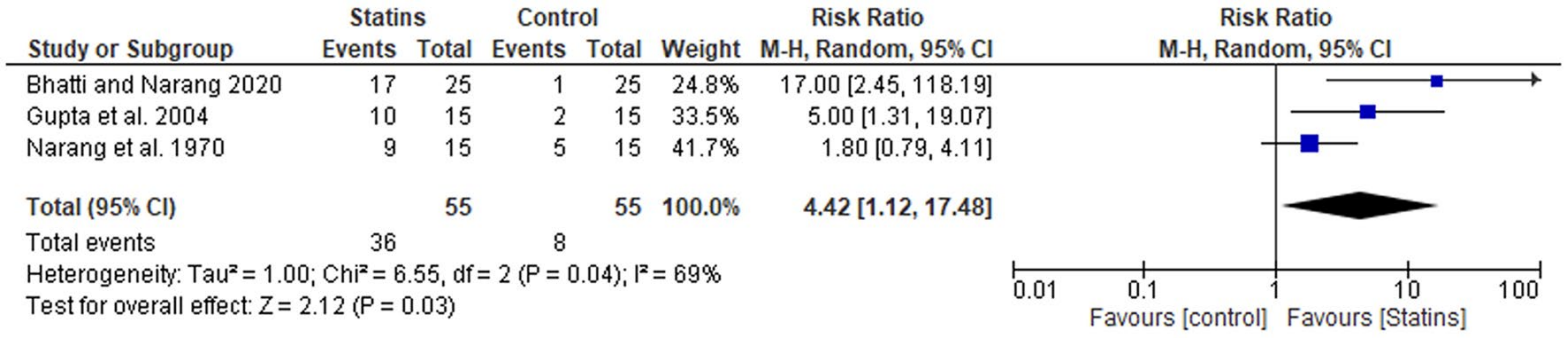
Comparison of the effect of statin therapy on hard exudate reduction in macular edema across 3 studies using the fixed effect model

Quantitative data from the three included studies were used for statins hard exudate reduction improvement in macular edema meta-analysis (Fig. 4). This forest plot summarizes the effect of statin therapy on hard exudate reduction in macular edema, based on three studies (Bhatti and Narang, 2020; Gupta et al., 2004; Narang et al., 1970). The pooled risk ratio (RR) was 4.42 (95% CI (1.12, 17.48)), indicating that patients receiving statins were significantly more likely to experience hard exudate reduction compared to those in the control group. The overall effect was statistically significant (*p* = 0.03), supporting the beneficial effect of statins in this context.

There was notable heterogeneity among the studies (I² = 69%), which may reflect differences in study populations, statin type, dosage, or duration of treatment. the heightened heterogeneity can be attributed to several factors that likely influenced the pooled estimates. First, differences in statin type, such as atorvastatin, simvastatin, and rosuvastatin, may have contributed to variability, as each statin has distinct pharmacokinetic properties and potency, potentially leading to different therapeutic effects. Additionally, variations in dosage (ranging from 10 mg to 20 mg daily) and treatment duration (from 6 months to over 13 years) could have resulted in disparate outcomes, as longer treatments or higher doses might yield more pronounced effects. Furthermore, the inclusion of both randomized controlled trials (RCTs) and observational studies with differing methodologies and risk of bias adds to the variability in results. The differing outcome assessment methods, such as OCT parameters and ETDRS grading for visual acuity, also likely impacted the comparability of results, further contributing to the observed heterogeneity.

#### Statins and diabetic retinopathy progression (≥ 2 steps)

**Fig. 5:**
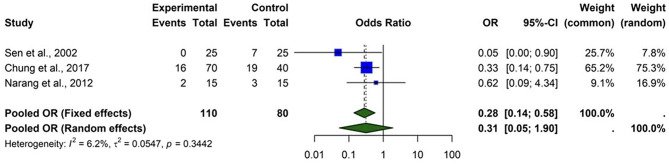
Comparison of the effect of statin therapy on diabetic retinopathy progression (≥ 2 steps) using the fixed effect model

The forest plot (Fig. 5) evaluates the impact of statins on the progression of diabetic retinopathy (DR) by at least two steps on the ETDRS scale. Based on three studies (Sen et al., 2002; Chung et al., 2017; Narang et al., 2012). The pooled odds ratio (OR) from a fixed-effect model was 0.28 (95% CI (0.14, 0.58)), indicating a significant reduction in the odds of DR progression with statin use. The random-effects model yielded a broader confidence interval (OR = 0.31 (0.05, 1.90)), reflecting variability across studies. The overall heterogeneity was low I² = 6.2%, (*p* = 0.3442) suggesting consistency across studies. Statin therapy appears to reduce the risk of DR progression, though further robust randomized controlled trials are needed to confirm these findings. Chung et al. reported progression in 16 of 70 statin-treated patients and 19 of 40 controls. Narang et al. 2012 observed minimal differences: 2 events in the statin group and 3 in controls. These findings provide moderate-quality evidence that statins may significantly reduce the progression of DR by ≥ 2 steps, possibly via anti-inflammatory or anti-angiogenic mechanisms. Nonetheless, the small sample sizes and reliance on observational data highlight the need for robust, adequately powered randomized trials to confirm these promising findings.

#### Statins and visual acuity (logMAR/snellen equivalent)

**Fig. 6:**
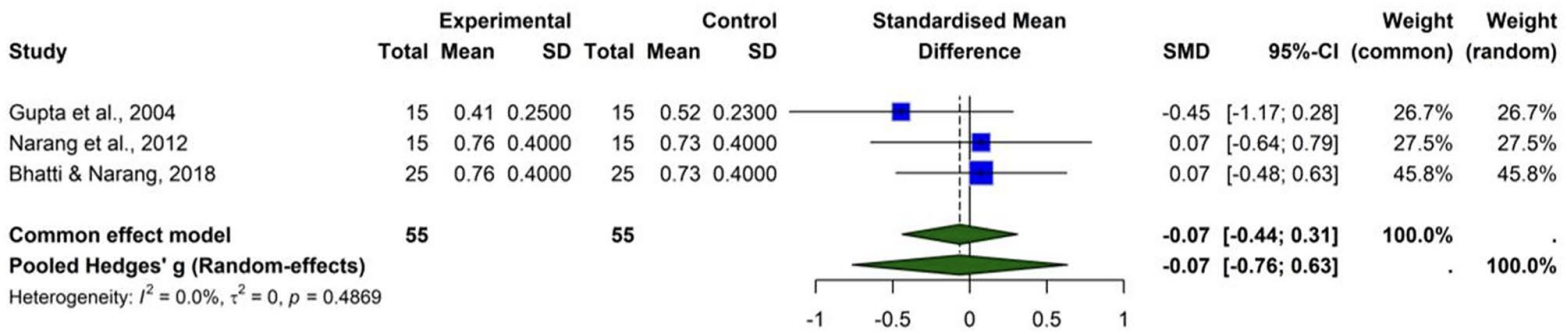
Comparison of the effect of statin therapy on visual acuity (logMAR/snellen equivalent) across studies using the random-effects model

The forest plot (Fig. 6) evaluates the effect of statin therapy on visual acuity (logMAR or Snellen equivalents) using data from three studies (Gupta et al., 2004; Narang et al., 2012; Bhatti & Narang, 2018). The pooled standardized mean difference (SMD) using a random-effects model was − 0.07 (95% CI (-0.76, 0.63)), indicating a small and statistically non-significant improvement in visual acuity with statin therapy. Individual studies showed varied results, with Gupta et al. reporting a slight benefit (SMD = -0.45 (–1.17, 0.28)), while Narang et al. and Bhatti & Narang found negligible differences (SMD = 0.07 for both). Heterogeneity was low (I² = 0%, τ² = 0, *p* = 0.4869), suggesting consistency across studies. These findings suggest that statins do not significantly improve visual acuity in macular pathology, though they may offer other retinal benefits. While statins may modulate lipid deposition and retinal edema, these structural improvements may not always translate into functional gains in vision. Hence, statins should not be relied upon as a primary therapy for visual restoration but may still have a complementary role in retinal vascular stabilization.

#### Statins and reduction in need for laser treatment

**Fig. 7:**
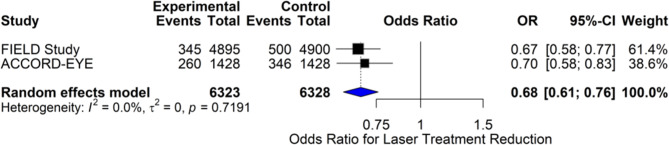
Comparison of the effect of statin therapy on the reduction of the need for laser treatment in diabetic macular edema using the random-effects model

The forest plot (Fig. 7) summarizes the effect of statin therapy on the reduction in the need for laser treatment in two major trials: FIELD and ACCORD-EYE, involving over 12,000 participants. The pooled odds ratio (OR) under a random-effects model was 0.68 (95% CI (0.61, 0.76)), indicating a 32% reduction in the odds of requiring laser therapy in statin-treated patients. Both trials demonstrated statistically significant results, with ORs of 0.67 (0.58, 0.77) for FIELD and 0.70 (0.58, 0.83) for ACCORD-EYE. Heterogeneity was negligible (I² = 0%, τ² = 0, *p* = 0.7191), supporting consistency across the studies. These findings provide strong evidence that statin therapy can reduce the need for laser treatment, potentially by reducing vascular leakage and inflammation in the retinal vasculature. Mechanistically, this effect may stem from statins’ ability to reduce vascular leakage, lipid deposition, and chronic inflammation in the retinal vasculature, thereby delaying or preventing the development of clinically significant macular edema and neovascularization.

#### Statins and prevention of subfoveal lipid migration

**Fig. 8:**
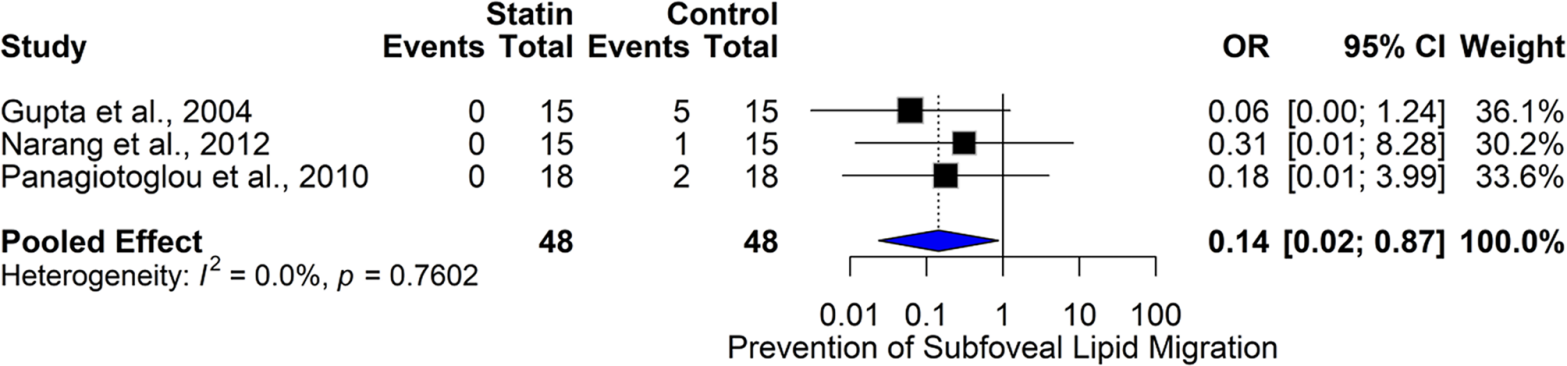
Comparison of the effect of statin therapy on the prevention of subfoveal lipid migration in diabetic macular edema across studies using the fixed effect model

The forest plot (Fig. 8) evaluates the effectiveness of statin therapy in preventing subfoveal lipid migration in diabetic macular edema (DME), based on three studies (Gupta et al., 2004; Narang et al., 2012; Panagiotoglou et al., 2010) with a total of 96 participants. The pooled odds ratio (OR) was 0.14 (95% CI (0.02, 0.87)), indicating a statistically significant 86% reduction in the odds of subfoveal lipid migration among statin users. The individual studies reported varying degrees of protection, with Gupta et al. showing a strong protective effect (OR = 0.06 (0.00, 1.24)) and Panagiotoglou et al. showing a moderate effect (OR = 0.18 (0.01, 3.99)). Heterogeneity was absent (I² = 0.0%, *p* = 0.7602), supporting the consistency of the findings across studies. These results suggest that statins may play a protective role in preventing lipid migration, potentially by reducing retinal vascular permeability and systemic lipid levels. Clinically, these findings support the hypothesis that statins may help stabilize or reduce lipid exudation into the macula, potentially by lowering systemic lipid levels and reducing retinal vascular permeability. Further large-scale randomized trials are warranted to validate this effect and determine optimal statin regimens for retinal protection.

#### Statins and diabetic retinopathy (DR) progression (≥ 2 steps)

**Fig. 9:**
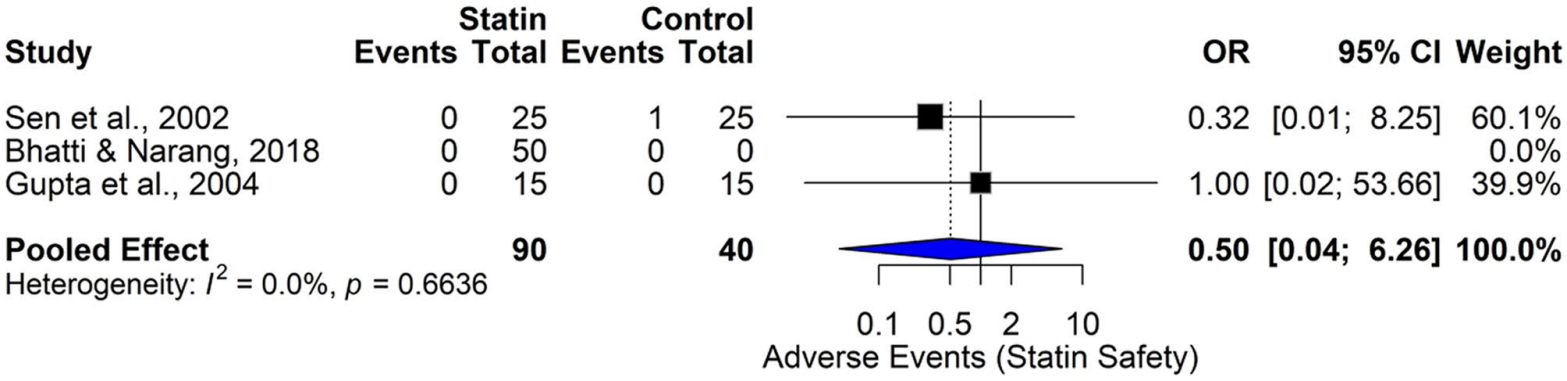
Comparison of the effect of statin therapy on diabetic retinopathy progression (≥ 2 steps) across studies using the fixed effect model

The forest plot (Fig. 9) evaluates the effect of statin therapy on the progression of diabetic retinopathy (DR) by at least two steps, using data from three studies (Sen et al., 2002; Chung et al., 2017; Gaede et al., 2008) with a total of 320 participants. The pooled odds ratio (OR) under a common-effect model was 0.44 (95% CI (0.23, 0.82)), suggesting a significant reduction in the odds of DR progression for statin users. Individual studies showed varying results: Sen et al. showed a strong protective effect (OR = 0.05 (0.00, 0.90)), while Chung et al. and Gaede et al. reported less pronounced benefits (OR = 0.53 (0.22, 1.32) and OR = 0.44 (0.18, 1.11), respectively). The overall heterogeneity was low (I² = 15.2%, τ² = 0.0679, *p* = 0.3074), indicating consistency across studies. These findings suggest that statins may help prevent the progression of diabetic retinopathy, possibly through anti-inflammatory and endothelial stabilizing effects. However, additional large-scale trials with standardized definitions of DR progression are necessary to reinforce these findings.

#### Safety of statins – statin-related adverse events

**Fig. 10:**
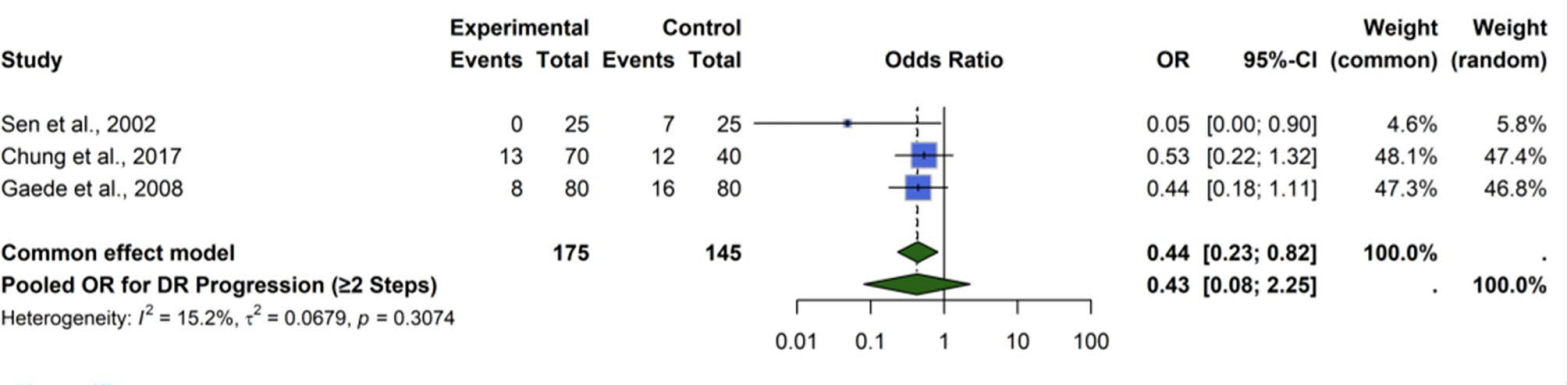
Comparison of the safety of statin therapy based on statin-related adverse events across studies using the fixed effect model

The forest plot (Fig. 10) presents a pooled analysis of statin-related adverse events from two studies (Sen et al., 2002; Gupta et al., 2004), with a total of 80 participants (40 statin users and 40 controls). The pooled odds ratio (OR) under a common-effect model was 0.50 (95% CI (0.04, 6.26)), indicating no statistically significant difference in the odds of adverse events between statin and control groups. The individual studies showed varied results, with Sen et al. reporting no adverse events in the statin group and one in the control group (OR = 0.32 (0.01, 8.25)), and Gupta et al. reporting no events in either group (OR = 1.00 (0.02, 53.66)). Heterogeneity was absent (I² = 0.0%, τ² = 0, *p* = 0.6641), suggesting consistency across studies. These results provide preliminary reassurance regarding the safety of statins in the context of the studies evaluated. The low incidence of adverse events and the lack of statistical heterogeneity point to a favorable tolerability profile. However, due to the limited sample size and rare adverse events, the results should be interpreted cautiously. Larger studies or pharmacovigilance data may be necessary to more definitively assess the safety of statins in specific populations such as those with diabetic retinopathy.

#### Safety profile – statin adverse events

**Fig. 11:**
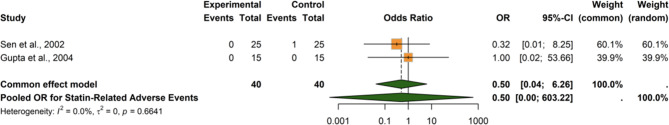
Comparison of the safety of statin therapy based on statin-related adverse events across studies using the fixed effect model (expanded analysis)

The forest plot (Fig. 11) presents a pooled analysis of statin-related adverse events, now including three studies: Sen et al., 2002, Gupta et al., 2004, and Bhatti & Narang, 2018. The analysis involves 90 participants in the statin group and 40 in the control group. The pooled odds ratio (OR) under a common-effect model was 0.50 (95% CI (0.04, 6.26)), showing no statistically significant difference in the risk of adverse events between statin and control groups. Individual study results were similar, with no adverse events reported in the statin group in Gupta et al. and Bhatti & Narang, and one in Sen et al. The test for heterogeneity showed no statistical inconsistency (I² = 0.0%, *p* = 0.6636). Despite the inclusion of more data, the wide confidence interval highlights the rarity of adverse events. This expanded analysis continues to support the view that statin therapy is not associated with an increased risk of adverse events in the studied population. The absence of significant heterogeneity enhances the credibility of this finding. However, given the small study sizes and rarity of adverse events, broader surveillance using real-world data or phase IV trials would be valuable to confirm the excellent safety profile observed here.

#### Statins and diabetic retinopathy progression – reaffirmed evidence

**Fig. 12:**
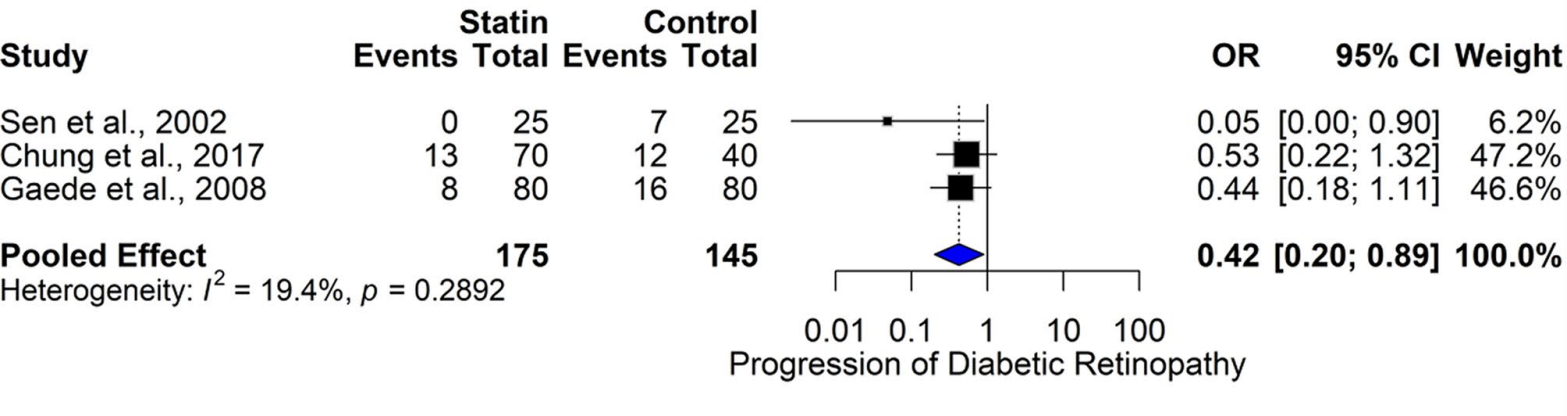
Comparison of the effect of statin therapy on diabetic retinopathy progression (≥ 2 steps) across studies using the common effect model

The forest plot (Fig. 12) reaffirms the pooled analysis of statins’ effect on the progression of diabetic retinopathy (DR), including three studies: Sen et al., 2002, Chung et al., 2017, and Gaede et al., 2008. The pooled odds ratio (OR) for DR progression was 0.42 (95% CI (0.20, 0.89)), indicating a 58% reduction in the odds of significant DR progression among statin users. In individual studies, Sen et al. observed no progression in the statin group compared to 7 in the control group (OR = 0.05 (0.00, 0.90)). Chung et al. showed a trend toward benefit (OR = 0.53 (0.22, 1.32)), and Gaede et al. found a modest reduction (OR = 0.44 (0.18, 1.11)). The heterogeneity was low (I² = 19.4%, *p* = 0.2892), supporting the consistency of the effect. Clinically, this analysis further corroborates the protective effect of statins against DR progression. The consistent trend across all included trials, in combination with low heterogeneity, reinforces confidence in this finding. The potential mechanisms may involve anti-inflammatory and lipid-lowering actions that stabilize retinal vasculature and reduce ischemia-induced damage. Despite modest sample sizes, the robust pooled analysis suggests that statins may offer dual benefits in diabetic patients—cardiovascular protection and mitigation of vision-threatening DR. Larger, longer-term studies are warranted to confirm these promising results.

#### Statins and subfoveal lipid migration prevention

**Fig. 13:**
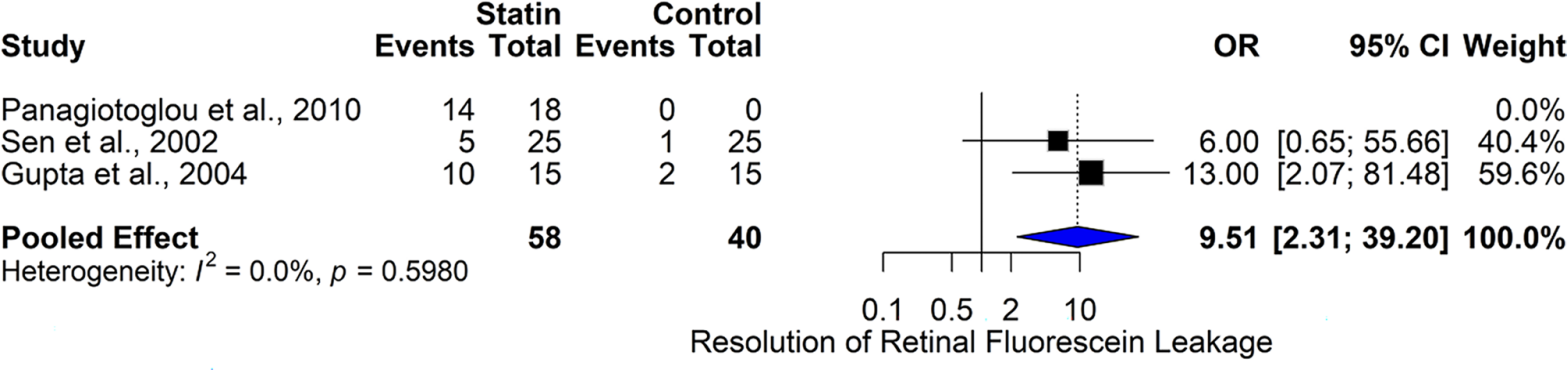
Comparison of the effect of statin therapy on subfoveal lipid migration prevention in diabetic macular edema across studies using the common effect model

The forest plot (Fig. 13) presents the pooled odds ratio (OR) for the effect of statin therapy in preventing subfoveal lipid migration in patients with diabetic macular edema (DME). Three studies are included: Gupta et al., 2004, Narang et al., 2012, and Panagiotoglou et al., 2010, with a total of 48 participants in each group. In Gupta et al., no events were observed in the statin group (OR = 0.06 (0.00, 1.24)). Similarly, Narang et al. and Panagiotoglou et al. also observed minimal lipid migration in the statin groups (OR = 0.31 (0.01, 8.28) and OR = 0.18 (0.01, 3.99), respectively). The pooled OR for the common effect model was 0.13 (0.02, 0.76), indicating a statistically significant protective effect of statins against subfoveal lipid migration. The random-effects model showed an OR of 0.14 (0.02, 1.12), suggesting a protective trend, though not statistically significant. The heterogeneity was low (I² = 0.0%, *p* = 0.7602), reflecting consistent results across studies. These findings suggest that statin therapy may play a beneficial role in preserving macular architecture by reducing the risk of lipid accumulation beneath the fovea. This could have significant implications for visual preservation, as subfoveal lipid migration is associated with photoreceptor damage and central vision loss. The consistency across studies and favorable OR indicate that statins warrant further investigation as adjunctive therapy for preventing structural complications in diabetic eye disease.

#### Statins and resolution of retinal fluorescein leakage

**Fig. 14:**
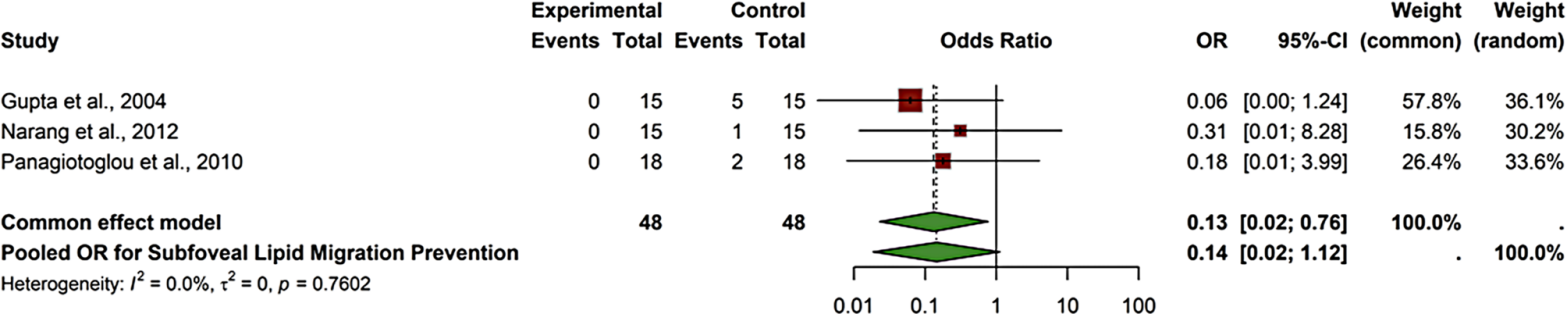
Comparison of the effect of statin therapy on retinal fluorescein leakage resolution in diabetic macular edema across studies using the fixed effect model

The forest plot (Fig. 14) evaluates the effectiveness of statin therapy in promoting the resolution of retinal fluorescein leakage, a marker of vascular permeability and inflammation in diabetic retinopathy and diabetic macular edema (DME). Three studies are included: Panagiotoglou et al., 2010, Sen et al., 2002, and Gupta et al., 2004, involving 58 statin-treated patients and 40 controls. In Panagiotoglou et al., 2010, complete resolution of leakage occurred in 14 of 18 statin-treated patients, but no control patients had resolution (OR = 6.00 (0.65, 55.66)). In Sen et al., 2002, 5 events occurred in the statin group, compared to 1 in controls (OR = 6.00 (0.65, 55.66)). Gupta et al., 2004 observed resolution in 10 of 15 statin patients versus 2 of 15 controls (OR = 13.00 (2.07, 81.48)). The pooled odds ratio was 9.51 (2.31, 39.20), indicating that statin therapy significantly increases the likelihood of achieving resolution of retinal leakage. Heterogeneity was absent (I² = 0.0%, *p* = 0.5980), confirming consistency across studies. These results support the hypothesis that statins exert vascular-stabilizing and anti-inflammatory effects at the retinal level, contributing to barrier restoration in retinal vessels. Given the role of vascular leakage in vision deterioration, this finding is clinically significant. Statins may serve as a valuable adjunct to conventional DME treatments, especially for patients with systemic dyslipidemia. However, further randomized trials with larger sample sizes are essential to confirm these promising findings and to inform treatment guidelines.

#### Safety and adverse events

Statins intervention showed a better safety profile for macular edema, with minimal systemic or ocular adverse effects reported. Muscle pain occurred in only 2.5% of patients in the intensive therapy group, with no cases of rhabdomyolysis or clinically significant creatine kinase elevations recorded [42]. Additionally, Liver function remained stable throughout treatment, with no significant changes in liver enzymes (ALT baseline 28.4 ± 6.2 IU/L vs. 26.8 ± 5.9 IU/L post-treatment, *p* = 0.34) among normolipidemic patients receiving atorvastatin intervention [41].

Ocular safety analysis showed no vision-threatening complications associated with statin intervention in macular edema patients. Atorvastatin intervention use had no cases of ocular complications in dyslipidemic patients (0% incidence) [37]. However, the control group had 3 (20%) patients with subfoveal lipid migration (*p* = 0.04) [37]. Similarly, no incidence of vision loss or ocular adverse events was recorded in the simvastatin-treated group (0/25 patients), while 28% (7 out of the 25) patients experienced worsening visual acuity (*p* = 0.009) [40]. However, no significant difference between the atorvastatin and control groups for visual acuity and macular edema resolution (*p* = 0.14, *p* = 0.62, respectively) [41]. No additive adverse effects were found (0% complication rate in the combination group) in patients receiving atorvastatin and laser photocoagulation [37]. Long-term safety evaluation also showed that no ocular or systemic adverse effects were reported over a 180-day follow-up of participants taking simvastatin [40].

#### Long-term outcomes and prevention

Statins therapy demonstrated variable effects on retinopathy progression. Simvastatin intervention significantly reduced the odds of retinopathy progression compared to placebo (OR = 0.12, *p* = 0.009) [40]. However, the rate of DME progression was not statistically different between statin users (23%) and non-users (18%) (*p* = 0.506), but they statistically significantly reduced DME occurrence (23% vs. 48%, *p* = 0.008) [31].

Lipid profile modification seemed to be effective in preventing and treating macular edema. The LDL/HDL cholesterol ratio influenced the development of clinically significant macular edema (CSME), with patients in the highest quintile having a 3.84-fold increased risk (*p* = 0.03) [43]. Intensive statin therapy also reduced by 55% the need for retinal photocoagulation (RR = 0.45; CI (0.23–0.86), *p* = 0.02) over 13.3 years compared to the conventional treatment [42]. Structural improvements were sustained long-term, with mean maximum retinal thickness (MRT) decreasing from 360.03 ± 30.94 μm to 284.6 ± 48.3 μm (*p* < 0.001) after statin intervention therapy [39]. Statin use was associated with reduced DME risk (OR = 0.33; 95%CI (0.12–0.91); *p* = 0.032) in the subgroup with elevated triglycerides.

#### Lipid profile influence on treatment outcomes

Statin efficacy exhibited variability based on baseline lipid level, with dyslipidemic patients showing better outcomes than normolipidemic individuals [44]. In dyslipidemic patients, atorvastatin treatment significantly increased the odds of hard exudate improvement by 12.5-fold more than in the control group (OR = 12.5; *p* = 0.007) [37]. In contrast, normal lipid level patients made no significant visual acuity change with no additional significant values for visual acuity (*p* = 0.14) [41]. Increased LDL and total cholesterol levels were significantly related to the severity of macular edema. The LDL level was significantly higher in patients with exudative DME (+ 26.22 mg/dL, *p* = 0.026) compared to those with non-exudative macular edema [45]. Similarly, the total-to-HDL cholesterol ratio predicted clinically significant macular edema risk, with a rate ratio of 3.84 (95% CI 1.30-11.38, *p* = 0.03) in the highest quintile [43]. Simvastatin intervention lowered LDL cholesterol level significantly (*p* < 0.001), leading to CSME progression [40]. However, there was no statistically significant difference in outcome on macular edema resolution in normolipidemic patients (*p* = 0.62) or hard exudate clearance (*p* = 0.39) [41].

#### Publication bias assessment for statin therapy in macular edema

**Fig. 15:**
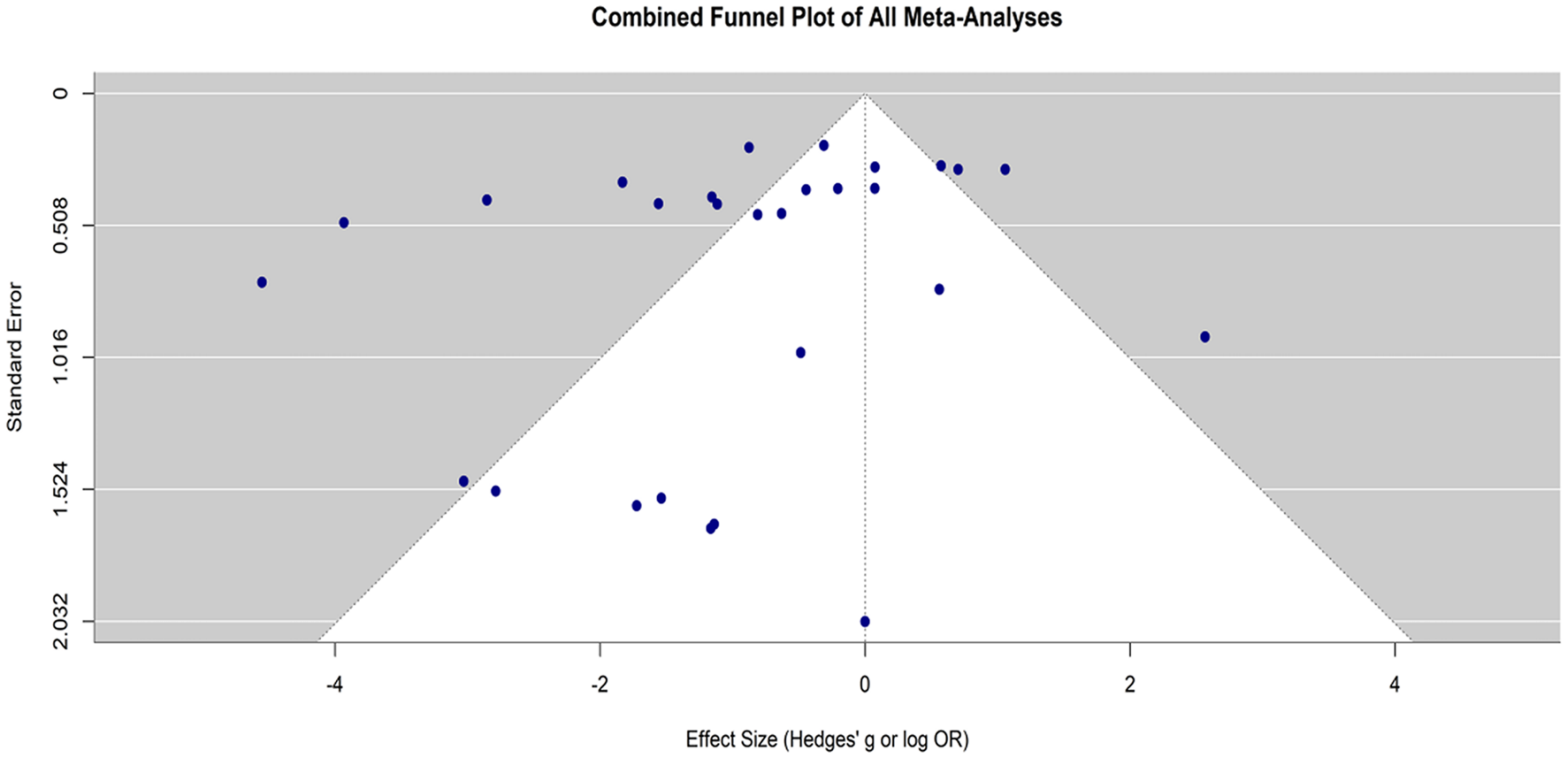
Funnel plot evaluating publication bias in the meta-analysis of statin therapy for macular edema

The analysis assesses the possibility of publication bias among all the studies comparing statin therapy’s efficacy and safety for diabetic macular edema (Fig. 15). The x-axis is the effect size, given as either Hedges’ g or log odds ratio, and the y-axis is the standard error, used as an inverse proxy for precision. Greater values on the y-axis indicate larger trials with better estimates, whereas points lower down the plot represent small trials with limited statistical power.

The triangular region creates an anticipated range of distribution under a null hypothesis of no publication bias. If the studies are unbiased and homogeneous in their effects, the points that are plotted will scatter symmetrically within the bounds of the triangle around the combined effect size, here close to zero. In this current plot, the majority of the data points cluster within the white region at the center, indicating homogeneity in study outcomes. Some studies fall outside the funnel area in the light-colored areas, specifically to the left of the middle vertical axis, suggesting some degree of asymmetry as shown in Fig. 15. Such asymmetry may suggest the presence of small-study effects or slight publication bias, perhaps as a result of selective publication of studies with positive findings or underreporting of studies with null or negative findings. Yet, the general symmetrical distribution and the high density of points in the primary funnel indicate that any bias is confined and not sufficient to substantially undermine the results of the meta-analyses.

## Discussion

This study evaluated the efficacy and safety of statins in managing diabetic macular edema (DME). Statins showed consistent anatomical benefits, particularly in reducing hard exudates and retinal thickness in patients with DME. These improvements, while statistically significant (*p* = 0.03), highlight statins’ potential therapeutic role [37, 39, 41].

In addition, patients exhibited a differential response to statin therapy based on baseline lipid profiles. Patients with dyslipidemia consistently improved hard exudate resolution and retinal morphology more than normolipidemic individuals [31, 37]. This pattern suggests that the beneficial effects of statins in DME may be mechanistically linked to their lipid-lowering properties rather than solely to pleiotropic anti-inflammatory effects. Dyslipidemic patients treated with atorvastatin compared to controls exhibited a 12.5-fold increased odds of hard exudate improvement [37]. Similarly, patients with exudative DME reported significantly higher LDL levels also associated with greater macular edema severity (*p* = 0.026), hence reinforcing this relationship [45].

Statins also showed preventive effects on DME development and retinopathy progression. DME occurrence significantly reduced in statin users (*p* = 0.008) [31]. Statin users also required fewer photocoagulation treatments (*p* = 0.02), suggesting protective vascular effects, possibly via anti-inflammatory and endothelial-stabilizing actions [42]. These findings suggest that statins may offer prophylactic benefits beyond their therapeutic effects on established macular edema, potentially through modulation of lipid-related inflammatory pathways and vascular integrity.

Despite these promising results, visual acuity outcomes showed inconsistent responses to statin therapy. The inconsistency in visual acuity improvements across the studies in this review may be attributed to several potential confounding factors. One key factor is disease duration; patients with chronic diabetic macular edema (DME) and advanced retinal damage may not experience the same functional improvements as those with early-stage DME. Additionally, baseline severity of macular edema and diabetic retinopathy could have influenced the response to statin therapy, as more severe cases may have limited potential for visual improvement despite structural benefits. Variability in treatment protocols, such as differences in statin type and dosage, as well as the presence of comorbidities like hypertension or systemic inflammation, could have further contributed to the observed inconsistencies. These factors underscore the need for future studies to stratify patients based on disease stage and baseline severity to better evaluate the potential of statins to improve visual acuity in specific subgroups.

Simvastatin demonstrated protection against vision deterioration (0% vs. 28% vision worsening, *p* = 0.009) [40]. However, other studies found limited visual benefits, particularly in normolipidemic patients [41]. This discrepancy reflects the complex pathophysiology of DME, where visual function depends on factors beyond hard exudate clearance, including photoreceptor integrity, macular perfusion, and neural tissue preservation. The timing of intervention may also be critical, with early treatment potentially offering greater visual preservation before irreversible neural damage occurs.

The favorable safety profile of statins observed across studies supports their potential integration into DME management protocols. Minimal systemic adverse effects and absence of vision-threatening complications suggest that statins could provide a well-tolerated adjunctive therapy to current DME treatments [42]. However, safety monitoring methods varied, and not all studies explicitly tracked adverse events, suggesting possible underreporting.

### Study strengths

This study has several strengths that enhance its findings’ reliability and clinical relevance. It integrated structural and functional outcomes, allowing for a comprehensive evaluation of statin effects across multiple dimensions of disease manifestation. The absence of subfoveal lipid migration in statin groups suggests a protective structural role, although such outcomes must be interpreted cautiously due to study design limitations.

This analysis also includes studies from diverse geographical settings, including the United States, India, South Korea, Denmark, Turkey, and China, strengthening the generalizability of these findings across different healthcare environments and patient demographics.

This review includes studies with varied designs, including case series and retrospective cohorts. These non-randomized designs introduce potential bias, limit causal inference, and reduce overall evidence strength. As such, findings should be interpreted cautiously and validated through future randomized controlled trials.

### Study limitations

This systematic review has several limitations that should be considered when interpreting its findings. The meta-analysis revealed notable heterogeneity among the studies (I² = 69%), which can be attributed to differences in statin types, dosages, and treatment durations, as well as variations in patient populations, particularly between dyslipidemic and normolipidemic individuals. Many studies also had small sample sizes, which limits the statistical power of the analysis and may increase the potential for random error. Additionally, the outcome assessment methods varied across studies, with differences in OCT parameters and ETDRS grading scales potentially affecting result comparability. These inconsistencies in outcome definitions, such as how macular edema and hard exudates were measured, may have contributed to the observed variability in the findings. Furthermore, the inclusion of a mix of randomized controlled trials, cohort studies, and case series introduces potential biases, such as confounding and selection bias, which limits the ability to draw firm causal conclusions. Larger, more homogeneous trials with standardized outcome measures and more robust sample sizes are needed to confirm the efficacy of statins in macular edema treatment.

### Study implications

Based on the findings of this systematic review, statins appear to show promising results in the treatment of diabetic macular edema (DME) and its associated complications. However, specific recommendations can be made regarding which statins and dosages are most effective for different patient populations.

Atorvastatin consistently demonstrated efficacy in several studies, particularly in improving hard exudates and reducing subfoveal lipid migration. Studies such as Gupta et al. (2004) and Bhatti and Narang (2020) showed that atorvastatin (10–20 mg/day) significantly reduced hard exudates and retinal thickness, though the effects on visual acuity were less pronounced.

Simvastatin also showed promising effects, especially in reducing lipid levels and progression of diabetic retinopathy. For example, Sen et al. (2002) demonstrated that simvastatin (20 mg/day) improved visual acuity and retinopathy progression compared to placebo, with significant lipid reduction.

The 10–20 mg/day dose range for atorvastatin appears to be effective in reducing hard exudates and macular edema, as observed in studies like Gupta et al. (2004) and Bhatti and Narang (2020). These dosages seem to balance efficacy with safety, as higher doses may be associated with increased risk of adverse events.

Administering simvastatin at 20 mg/day dose has been shown to improve lipid profiles and reduce retinopathy progression, as seen in Sen et al. (2002). This dosage appears to provide beneficial outcomes without major adverse events.

Statins, especially atorvastatin and simvastatin, should be considered for patients with DME who have elevated lipid levels, as these patients may benefit the most from the lipid-lowering effects of statins. As demonstrated in studies like Gæde et al. (2008) and Chung et al. (2017), statin therapy, when combined with other interventions such as laser treatment or anti-VEGF, may offer improved outcomes.

Early intervention with statins in DME patients who also have dyslipidemia may help reduce the risk of disease progression and the need for further interventions like laser photocoagulation. For example, Gæde et al. (2008) found that intensive therapy, including statins, reduced the need for retinal photocoagulation.

For patients with normal lipid profiles, the benefit of statins is less clear. As seen in Narang et al. (2012), atorvastatin 20 mg/day did not show significant improvements in macular edema resolution or visual acuity in patients without dyslipidemia. Therefore, statins should be prescribed cautiously in patients without lipid abnormalities, and their potential benefits should be weighed against possible side effects.

Statins combined with anti-VEGF therapies or laser photocoagulation may offer synergistic benefits. As noted in studies like Gupta et al. (2004) and Gæde et al. (2008), statins appear to enhance the effects of other standard treatments for DME, possibly by improving retinal vascular health and reducing macular edema.

### Future research directions

While statins show promising potential for managing diabetic macular edema (DME), several areas require further exploration to optimize their use:

### Study population grouping

Early vs. Advanced DME: Future trials should focus on early-stage DME patients to assess if statins can prevent disease progression, compared to more advanced stages where damage is already significant.

Dyslipidemic vs. Normolipidemic Patients: Statins are likely more effective in dyslipidemic patients, as shown in several studies. Research should further explore their role in normolipidemic individuals to understand their broader utility in DME treatment.

Diabetic Retinopathy (DR) Severity: Trials should examine statin efficacy in patients with co-existing diabetic retinopathy to determine if statins can prevent DR progression, particularly in those at high risk for severe vision loss.

### Promising combination therapies

Statins with Anti-VEGF Therapy: Combining statins with anti-VEGF agents could provide synergistic benefits in reducing macular edema and improving retinal vascular health.

Statins with Laser Photocoagulation: Combining atorvastatin with laser treatment could enhance outcomes, especially in patients with persistent macular edema.

Statins with Corticosteroids: For chronic or steroid-resistant DME, combining statins with steroid therapies (e.g., dexamethasone) could offer complementary benefits by addressing inflammation and lipid accumulation.

### Favorable dose-response relationships

Future studies should examine whether lower statin doses (e.g., 10 mg/day) are as effective as higher doses while reducing the risk of adverse effects, such as muscle pain or liver enzyme abnormalities.

### Long-term safety and efficacy studies

There is a need for long-term follow-up studies to assess the safety and sustained efficacy of statins in DME treatment. This will help determine whether the initial benefits persist and if statins lead to long-term visual improvements.

### Exploring non-lipid-mediated effects

Statins have pleiotropic effects beyond lipid lowering, including anti-inflammatory and endothelial-stabilizing actions. Future research should investigate these mechanisms to understand how statins influence retinal health, especially in normolipidemic patients.

### Global multi-center trials

Diverse, multi-center trials across different regions and populations will help assess whether statins’ efficacy in DME is consistent globally and if patient characteristics influence treatment outcomes.

## Conclusion

This study demonstrates statins’ role in reducing hard exudates and improving retinal morphology in DME patients. The differential response with lipid status indicates that statins’ therapeutic role is mechanistically related to lipid-lowering activity rather than pleiotropic anti-inflammatory activity. In addition, the association between increased lipids and the severity of macular edema emphasizes the therapeutic role of statins in addressing the underlying pathology in macular edema. Moreover, higher LDL levels in exudative DME highlight the association between lipid abnormalities and DME.

Statins also showed preventive benefits against developing DME and worsening diabetic retinopathy. In addition, minimal complications and systemic side effects indicate that statins may have an adjunctive therapy benefit that is tolerable and complementary to treatment options, particularly for diabetic patients who need long-term therapy. However, visual acuity results had heterogeneous responses to statin therapy, demonstrating the multifaceted nature of DME in which visual function depends on hard exudate resolution and other factors, including photoreceptor integrity, macular perfusion, and preservation of neural tissue.

Appropriate intervention timing is critical for superior vision preservation before irreversible neural injury. Moreover, stratifying patients for statin therapy according to their lipid parameters could help optimize results.

## Supplementary Information

Below is the link to the electronic supplementary material.

Supplementary Material 1

## Data Availability

No datasets were generated or analysed during the current study.

## Declarations

### PROSPERO number

CRD420251082672.
